# Cytokine production by human B cells: role in health and autoimmune disease

**DOI:** 10.1093/cei/uxac090

**Published:** 2022-09-30

**Authors:** Nina M de Gruijter, Bethany Jebson, Elizabeth C Rosser

**Affiliations:** Centre for Adolescent Rheumatology Versus Arthritis at University College London, University College London Hospital and Great Ormond Street Hospital, London, UK; Centre for Rheumatology Research, Division of Medicine, University College London, London, UK; Centre for Adolescent Rheumatology Versus Arthritis at University College London, University College London Hospital and Great Ormond Street Hospital, London, UK; University College London Great Ormond Street Institute of Child Health, London, UK; Centre for Adolescent Rheumatology Versus Arthritis at University College London, University College London Hospital and Great Ormond Street Hospital, London, UK; Centre for Rheumatology Research, Division of Medicine, University College London, London, UK

**Keywords:** B cells, cytokine, inflammation, autoimmunity

## Abstract

B cells are classically considered solely as antibody-producing cells driving humoral immune responses to foreign antigens in infections and vaccinations as well as self-antigens in pathological settings such as autoimmunity. However, it has now become clear that B cells can also secrete a vast array of cytokines, which influence both pro- and anti-inflammatory immune responses. Indeed, similarly to T cells, there is significant heterogeneity in cytokine-driven responses by B cells, ranging from the production of pro-inflammatory effector cytokines such as IL-6, through to the release of immunosuppressive cytokines such as IL-10. In this review, focusing on human B cells, we summarize the key findings that have revealed that cytokine-producing B cell subsets have critical functions in healthy immune responses and contribute to the pathophysiology of autoimmune diseases.

## Teaser

B cells are classically considered solely as antibody-producing cells driving humoral immune responses to foreign antigens in infections and vaccinations as well as self-antigens in pathological settings such as autoimmunity. However, it has now become clear that B cells can also secrete a vast array of cytokines, which influence both pro- and anti-inflammatory immune responses. In this review, focusing on human B cells, we summarise the key findings that have revealed that cytokine-producing B cell subsets have critical functions in healthy immune responses and contribute to the pathophysiology of autoimmune diseases.

## Graphical Abstract

**Graphical Abstract:**
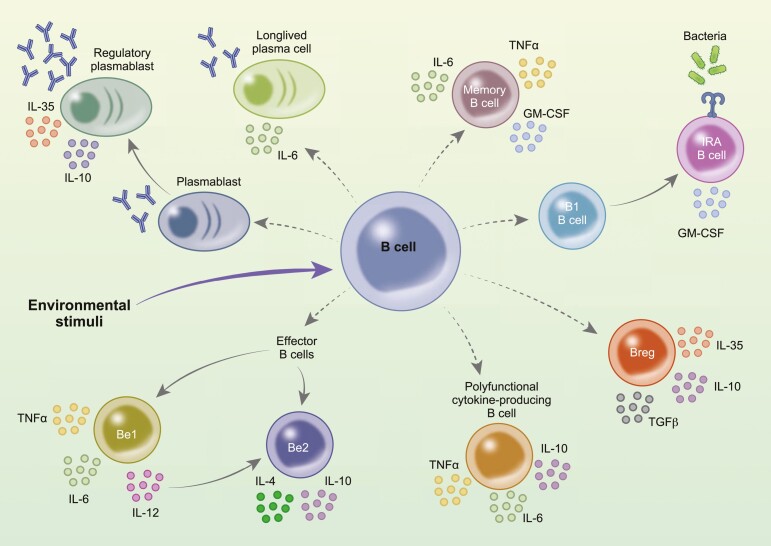

## Introduction

**Figure 1:**
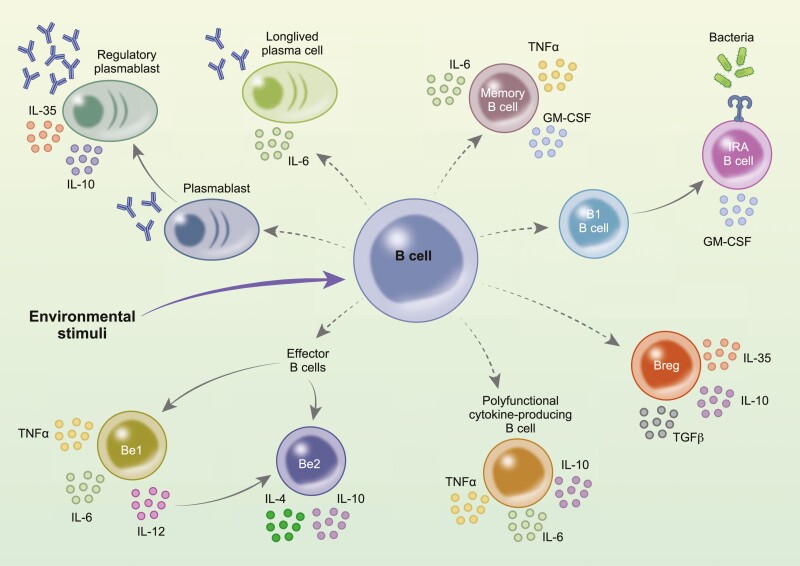
polyfunctionality of cytokine-producing B cell subsets. The schematic summarises the currently described functional subsets of cytokine-producing B cells. IL: interleukin; TNF: tumour necrosis factor; IFN: interferon; LT: lymphotoxin; GM-CSF: granulocyte-macrophage colony-stimulating factor. Breg: regulatory B cell.

The life cycle of a B cell starts in the bone marrow (BM), where haemopoietic stem cells rearrange the heavy chain and light chain loci to produce a functioning B cell receptor (BCR) [1–4]. In humans, immature B cells then exit the BM as transitional B cells (CD19^+^CD24^hi^CD38^hi^) before they mature in the periphery as naïve B cells (CD9^+^CD24^int^CD38^int^) [1–4]. Upon recognition of antigen, mature naïve B cells can further differentiate into non-switched or switched memory B cells (CD19^+^CD24^hi^CD38^lo^CD27^+^), short-lived plasmablasts (CD19^+^CD24^lo^CD38^+^CD27^+^), and long-lived plasma cells (CD19^+/lo^CD38^+^CD27^+^CD138^+^) [1–4]. Along with the BM-derived B cells, similarly to mice, humans are also thought to possess a subset of B cells known as B-1 cells. Innate-like B-1 cells have been shown to be formed during foetal and neonatal development and are a key player in the first-line defence against pathogens [5]. These cellular differentiation steps are well recognized for their involvement in effective humoral immune responses by leading to the production of high affinity antibodies to new and—through the generation of immunological memory—historical antigens. However, research conducted over the last 40 years has revealed that B cell subsets at various stages of maturation also have the capacity to produce a wide array of cytokines. The induction of cytokine-producing B cells occurs following exposure to similar stimuli that are needed to induce antibody production, including activation of CD40, toll-like receptors (TLRs), and/or sequential linking of the BCR [6–10]. The ultimate effector function of cytokine-producing subsets is context dependent and highly influenced by the surrounding cytokine micro-environment. The complex array of cytokines produced by human B cells (Fig. 1), which is the subject of this review, is now appreciated to play a critical role in modulating a broad range of immune responses.

In this review, we provide a consolidated view of the current understanding of the human cytokine-producing B cell field. We describe the initial observations that launched the study of cytokine production by human B cells and summarize the evidence that cytokine production by human B cells is context dependent and can be broadly subdivided into two subsets: cytokine-producing B cells that propagate and those that regulate immune responses. We discuss data demonstrating that this system is both highly dynamic and polyfunctional, as, unlike in T cells, discrete cytokine profiles cannot be assigned to discrete B cell subsets defined by the expression of specific transcription factors. We finally highlight key studies using samples from patients with autoimmune diseases to demonstrate the pathological impact of dysfunctional cytokine production by human B cells. These data are particularly important when considering the increasing use of B cell depletion therapies (BCDT) to treat a broad range of autoimmune conditions including systemic lupus erythematosus (SLE) and rheumatoid arthritis (RA), as the efficacy of BCDT is highly likely to be influenced by the modulation of cytokine-producing subsets of B cells. This review aims to highlight key findings that have established that B cells contribute to immune homeostasis and inflammation through pleiotropic effector functions that are not solely dependent on their ability to produce antibodies.

### The 1980s and the launch of a new field: cytokine production by human B cells

**Figure 2:**
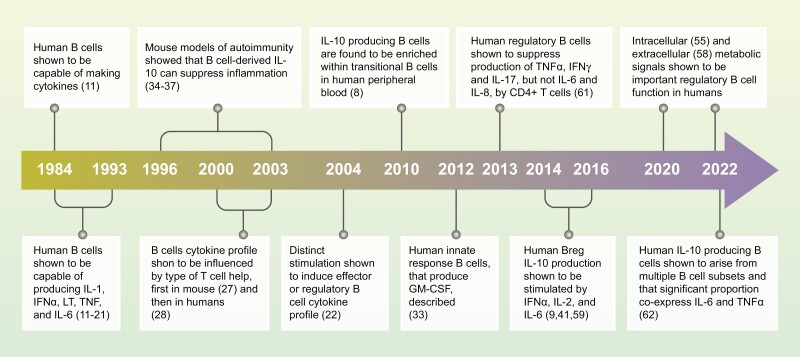
a timeline of key discoveries characterising the function of cytokine-producing B cell subsets in humans. The ability of B cells to secrete cytokines was first described in the 1980s and 1990s. These studies formed the basis of the field as it stands today: that context-dependent activation of human B cells leads to the production of both pro- and anti-inflammatory cytokines and that production of these cytokines is altered in different pathologies. IL: interleukin; TNF: tumour necrosis factor; IFN: interferon; LT: lymphotoxin; GM-CSF: granulocyte-macrophage colony-stimulating factor.

The understanding that human B cells can produce cytokines is not a recent development in immunological research. Evidence from as early as 1984 showed that, following appropriate stimulation, human B cells could produce cytokines [11]. By 1990 studies had shown (mainly leukemic and/or Epstein-Barr Virus (EBV)-transformed) B cells to produce interleukin (IL)-1 [11–13], interferon (IFN)α [14], lymphotoxin (LT; also known as TNFβ) [15, 16], tumor necrosis factor (TNF) [17], and IL-6 [18, 19]. Studies in the 1990s were critical in establishing that cytokine production by human B cells is an important part of their function, not only in situations of B cell stress—i.e. EBV transfection or malignancy—but also in normal immunological responses [6, 20, 21]. In 1991, Rieckmann and colleagues showed that both tonsil and peripheral blood (PB)-derived human B cells can produce TNFα and IL-6 when stimulated with a combination of IL-2 and *Staphylococcus aureus* Cowan strain I (SAC)—a polyclonal B cell activator that works by cross-linking of the BCR [21]. This study also investigated how cytokine production by PB B cells was affected by hypergammaglobulinemia—i.e. high antibody titres in the blood due to HIV infection or autoimmune conditions (SLE, RA, or Wegner’s granulomatosis) [21]. B cells from individuals with these conditions produced significantly higher levels of TNFα and IL-6 than PB or tonsillar B cells from healthy individuals, even when cultured without *in vitro* stimulation [21]. In 1993, Matthes and colleagues used healthy PB B cells—co-cultured with mouse thymoma cells expressing CD40 ligand (CD40L) and/or supernatant from stimulated human T cells to simulate human T cell help [20]—to demonstrate that activated human B cells can produce TNFα, IL-6, tumor growth factor (TGF)β and IL-10. Finally, in 1995, Burdin and colleagues reported that activated tonsillar B cells could produce a wide range of cytokines, whilst confirming that non-activated B cells from healthy individuals do not spontaneously produce cytokines, except for very low amounts of IL-6 [6]. In this study, activated B cells produced IL-1β, IL-6, IL-10, granulocyte-macrophage colony-stimulating factor (GM-CSF) and TNF. Importantly, the authors noted that the amount of each cytokine produced was context-dependent, with BCR crosslinking (by SAC or anti-μ) or CD40 activation inducing different cytokine profiles [6]. Collectively, these early studies established the basic concepts that form the basis of the human cytokine-producing B cell field as it stands today (**Fig. 2**).

### Context-dependent production of cytokine by B cells: propagation of immune responses

The seminal study by Duddy and colleagues in 2004 formed the second key observation that human B cell cytokine production is context-dependent, with B cells producing different cytokine profiles in response to different stimuli [22]. The authors showed that when human B cells were activated via BCR cross-linking and CD40 stimulation, they produced effector cytokines IL-6, TNFα and LTα [22]. When B cells received only CD40 stimulation, they produced the immunoregulatory cytokine IL-10 and significantly lower levels of effector cytokines [22]. Importantly, this cytokine network was dysfunctional in patients with multiple sclerosis (MS), leading to enhanced IL-6 production and a reduction in IL-10 production [22, 23]. Multiple studies have now described the potential importance of B cell-derived IL-6 in propagating immune responses in humans. More specifically, in mice, B cell derived IL-6 has been shown to influence a wide variety of T cell subsets. Examples include supporting Th17/Th1 differentiation in a model of tuberculosis infection and forming a positive feedback loop to induce antibody production supporting T follicular helper cell differentiation and thus GC formation [24, 25]. Accordingly, although not directly attributed to B cells alone, in numerous autoimmune diseases, such as MS, SLE and RA, serum levels of IL-6 have correlated with disease activity and autoantibody titres [26]. These data suggest that there may be a positive feedback loop between IL-6-producing B cell subsets and autoantibody-producing B cell subsets.

Also in the early 2000s, Frances Lund’s group performed seminal work using mouse B cells to show that B cells could secrete a wider array of cytokines than previously appreciated and that this could happen in a context-dependent manner [27]. Specifically, the authors showed that B cells could differentiate into effector cells with distinct cytokine profiles known as B effector (Be)1 and Be2. In the presence of CD4^+^ T helper (Th)1 cell, Be1 cells released inflammatory Th1-like cytokines such as IFNγ, TNFα and IL-12, and in the presence of Th2-like cells, Be2 cells released type 2 cytokines such as IL-4 and IL-10 [27]. Although these studies were conducted using samples from mice, they were the first to suggest that—similarly to other antigen-presenting cells such as dendritic cells and macrophages—B cells can influence the nature of a generated immune response by modulating the cytokine micro-environment. Importantly, there is evidence that human B cells have the potential to differentiate into Be1 and Be2 subsets. Using a human B cell line, Durali and colleagues showed that when B cells were cultured with Th1 cells, IL-12 released by the Th1 cells caused signal transducer and activator of transcription 4 (STAT4) activation and subsequent IFNγ release by B cells or Be1 phenotype [28]. Around the same time, Flaishon and colleagues also showed that immature B cells autocrinally prevent their premature entry to lymph nodes and exposure to antigen, by expressing IFNγ and CCL2, which downregulate their integrin-mediated adhesion to the extracellular matrix [29–31].

When considering context-dependent production of effector cytokines, different B cell subsets are potentially predisposed to produce certain cytokines. For example, following stimulation through BCR cross-linking and CD40L-expressing T cells *in vitro*, human memory B cells (CD19^+^CD27^+^) release both LT and TNFα, which may promote organogenesis and subsequent germinal centre formation [23]. Complementarily, Li and colleagues identified a subset of memory B cells expressing high levels of GM-CSF, along with substantial amounts of pro-inflammatory cytokines IL-6 and TNFα, and found that this subset was expanded in MS patients [32]. Notably, GM-CSF production has been attributed to a subset of B-1-like cells termed ‘innate response activator’ (IRA) B cells. These IRA B cells play an important role in the first line of defence against infection and have been identified in the human spleen, cord blood and pleural cavities [33]. Whether GM-CSF production by memory B cells also has a role in pathogen clearance is unclear. However, these data demonstrate that multiple inflammatory cues, both autoreactive and infectious, can upregulate pro-inflammatory cytokine production by B cells.

### Identification of cytokine-producing B cells that suppress immune responses: regulatory B cells

An important concept raised by the early studies discussed above was that under different conditions, human B cells can produce anti-inflammatory cytokines or pro-inflammatory cytokines. Accordingly, a regulatory or suppressive role for cytokine production by human B cells has been the focus of intense study over the last decade and is an expanding niche in B cell research. Although regulatory B cells or Bregs are now well characterized in humans, studies from the late 1990s and early 2000s, using mouse models of autoimmunity, formed the basis of the human regulatory B cell field as we know it today. In 1996, Wolf and colleagues showed that B cell-deficient mice had similar disease onset as wildtype mice in spontaneous experimental autoimmune encephalitis (EAE) (B10.PL; a model of multiple sclerosis), but significantly worse disease recovery [34]. Fillatreau and colleagues used a bone marrow chimeric system of myelin oligodendrocyte glycoprotein (MOG)-induced EAE, in which only B cells were IL-10 deficient, to show that IL-10 production by B cells was required for disease recovery [35]. That same year, Mizoguchi and colleagues showed that IL-10-producing B cells could suppress the progression of existing disease in a mouse model of colitis (TCRαKO) [36]. In the early 2000s, Mauri and colleagues demonstrated IL-10-producing B cells were also able to suppress experimental arthritis [37]. Building on this work in the 2010s, the same group used chimeric mice lacking IL-10-producing B cells to show that IL-10 from B cells regulates arthritis inflammation by suppressing Th17/Th1 responses and inducing T regulatory type I cells [38, 39].

In humans, the existence of a regulatory B cell subset was first confirmed by Blair and colleagues in 2010 [8]. This seminal paper demonstrated that IL-10-producing B cells are enriched in the CD19^+^CD24^hi^CD38^hi^ transitional B cell population and could suppress pro-inflammatory T cell responses *in vitro* [8]. Human regulatory B cells have since been ascribed several other distinct phenotypes including CD24^hi^CD27^+^ [10], CD73^-^CD25^+^CD71^+^ [40], CD27^int^CD38^+^ [41], CD19^+^CD5^+^ [42], CD19^+^CD27^+^IgA^+^ [43] and CD19^+^CD27^-^IgD^+^IgM^+^CD24^hi^CD38^hi^CD1d^hi^ [7]. Although IL-10 production is the most common—and most commonly studied—regulatory mechanism of Bregs, human Bregs can also suppress immune responses via the production of other anti-inflammatory cytokines [44, 45], including TGFβ [46] and IL-35 [47], or through the expression of surface-bound inhibitory molecules, such as programmed death-ligand 1 (PD-L1) [43].

In mice, innate-like populations of B cells, such as marginal zone B cells and B-1 cells can also produce IL-10 [48, 49]. Research into these subsets in humans has been complicated by limited access to the lymphoid tissues and peritoneal fluid, the predominant sites at which innate-like B cells can be found in mice. However, CD11b^+^ B-1 cells that spontaneously produce IL-10 have been recently described in human peripheral blood [50]. It is likely that with the increased availability of single cell transcriptomic data sets analysing human lymphoid tissue, IL-10-producing populations of innate-like B cells will become better defined in the next few years [51].

### Induction and function of regulatory B cells in humans

The exact signals needed to induce human B cells to become a Breg *in vivo* remain unknown, but factors that induce IL-10 production by human B cells *in vitro* are well defined [52, 53]. Indeed, key signals that induce IL-10 production by human B cells overlap with those described in mice and include activation of CD40 [7, 8] and toll-like receptors like TLR9 [9, 10]. Specifically, Liu and colleagues showed that activation of TLR7/8 or TLR9 on human B cells caused phosphorylation of extracellular signal-regulated kinases (ERK) and signal transducer and activator of transcription 3 (STAT3), which led to IL-10 production by B cells [9]. More recently, CD40L-expressing type 3 innate lymphoid cells (ILC3s) were shown to induce B cell IL-10 production [7]. Simultaneous TLR signaling may be required for this effect, as another study showed that B cells produced IL-10 when stimulated with soluble CD40L *in vitro*, but only in the presence of TLR agonists [9]. Notably, the co-cultures in the ILC3-B cell experiment were initially stimulated with CpG—a potent TLR agonist—which not only acted directly on the B cells, but also increased soluble CD40L production by ILC3s, which express TLR9 [7]. Human Bregs also express receptors that allow for negative regulation, such as SLAMF5, to maintain Breg homeostasis. Blocking SLAMF5 increased the Breg population when human PB B cells were stimulated *in vitro*, and induced transcription of *maf*, a gene required for IL-10 production [54].

Bibby and colleagues have also shown that metabolic priming events—driven by cholesterol metabolites and leading to geranylgeranyl pyrophosphate (GGPP) synthesis—are necessary to induce IL-10 production following stimulation with CpG [55]. GGPP regulates IL-10 production by human B cells by modulating signalling downstream of TLR9-activation via PI3Kδ-AKT-GSK3, ultimately leading to upregulation of the transcription factor BLIMP-1 [55]. This study was one of the first to uncover the molecular mechanisms driving IL-10 production—and indeed cytokine production—by human B cells. However, this has not been the only study to demonstrate that metabolic stimuli are associated with the induction of immunosuppressive cytokine secretion by B cells. The levels of microbially-derived metabolites or short-chain fatty acids butyrate and acetate are associated with the production of IL-10 by CD19^+^CD24^hi^CD38^hi^ transitional B cells [56] and CD24^hi^CD27^+^ B10 cells [57]. It was also recently suggested that diets rich in the essential amino acid leucine drive the TGF-β production in leucine-tRNA-synthase-2-expressing (LARS) B cells [58]. These cells may have a role in supporting immune evasion of tumour cells in colorectal cancer (CRC), contributing to adverse outcomes in CRC patients [58].

Several proteins, including cytokines, contribute to inducing IL-10 production by Bregs. IFNα—*in vivo* often derived from plasmacytoid dendritic cells (pDCs)—is well known to support Breg differentiation and production of IL-10 [9, 41, 59]. Matsumoto and colleagues showed that the presence of IL-2, IL-6, and IFNα significantly increased the IL-10 production by human B cells upon stimulation with CpG *in vitro* [41]. A proliferation-inducing ligand (APRIL)—a member of the TNF family that is known to stimulate B cells [60]—can also induce IL-10 production by B cells. Hua and colleagues showed that the presence of APRIL significantly increased B cell IL-10 production in a peripheral blood mononuclear cell (PBMC) culture that was stimulated with CpG [60]. Furthermore, Fehres and colleagues showed that stimulating naïve B cells with a combination of APRIL and IL-21 promoted the differentiation (by class-switching) of an IgA^+^ Breg subset and induced IL-10 secretion [43]. Though the presence of IL-21 was required to drive proliferation and subsequent IL-10 production, stimulation with IL-21 or APRIL alone did not significantly induce Breg IL-10 production [43]. Interestingly, B cell activating-factor (BAFF)—another B cell-stimulating member of the TNF family—does not seem to consistently induce Breg IL-10 production, though it has been tested repeatedly [7, 42, 43, 60]. For example, the presence of BAFF did not influence Breg IL-10 production in a CpG-stimulated PBMC culture compared to CpG alone [60]. Blocking BAFF receptor neither influenced B cell survival nor IL-10 production in the CD40L^+^ ILC3-B cell co-cultures described above [7]. BAFF also did not promote the differentiation of naïve B cells into IgA^+^ IL-10 producing Bregs, regardless of the presence of IL-21 [43]. One report did describe an increase in IL-10 production when comparing stimulation of CD19^+^CD5^+^ B cells with BAFF+CpG to CpG alone—an effect which was attenuated by blocking the BAFF receptor [42].

Following activation, Bregs exert their suppressive effect on immune responses via several mechanisms. Blair and colleagues showed that Bregs suppress the production of pro-inflammatory cytokines TNFα and IFNγ by CD4^+^ effector T cells [8]. They co-cultured CD40L-stimulated CD19^+^CD24^hi^CD38^hi^ B cells—which contain the majority of the Breg population—and CD4^+^CD25^-^ T cells, and showed that these T cells produced significantly lower levels of TNFα and IFNγ when stimulated with anti-CD3 than T cells co-cultured with other CD40L-stimulated B cell subsets [8]. Breg-derived IL-10 was (at least partially) responsible for this effect, as neutralization of IL-10 restored TNFα production by the effector T cells, and partially restored IFNγ production [8]. Flores-Borja and colleagues used a similar system to confirm that CD19^+^CD24^hi^CD38^hi^ B cells suppress the production of type 1 (TNFα and IFNγ) and type 17 (IL-17) cytokines by CD4^+^ T cells, but that their IL-6 and IL-8 production was not affected [61]. They further showed that CD19^+^CD24^hi^CD38^hi^ B cells play an important role in T cell homeostasis: they maintain a regulatory T cell (Treg) pool and support Treg differentiation by increasing the expression of Treg transcription factor *foxp3* in naïve CD4^+^ T cells [61]. Hua and colleagues used a different co-culture system to confirm a suppressive effect of Bregs on CD4^+^ T cells: they stimulated B cells with APRIL to induce a regulatory phenotype before co-culturing them with CD4^+^ T cells [60]. Compared to unstimulated B cells, the APRIL-stimulated B cells significantly reduced the percentage of CD4+ T cells that were TNFα^+^ and IFNγ^+^, but not IL-17^+^ [60]. Notably, Bregs also exert an influence on leukocyte populations in the innate arm of the immune system, such as monocytes and pDCs. Following stimulation with CD40L and CpG, CD24^hi^CD27^+^ B cells suppressed TNFα production by CD4^+^ T cells or monocytes in an IL-10-dependent manner [10], whilst CD40L-stimulated CD24^+^CD38^hi^ Bregs suppressed production of IFNα by pDCs via IL-10 as well [59].

A recent conundrum in the Breg field is the fact that the same signals that induce IL-10 production by B cells can also induce the production of antibodies [41, 53]. Potential theories for this dichotomy include that downstream effects depend on the phenotype of the B cell receiving the signals, as it appears memory B cells—unlike CD24^hi^CD38^hi^ immature B cells [59]—cannot develop into IL-10-producing plasma cells upon stimulation *in vitro* [41]. The concentration of stimulants in the local environment could also be a factor, as Menon and colleagues showed that the concentration of IFNα determined whether an immature B cell became a Breg or a plasmablast *in vitro* [59]. However, this hypothesis is complicated by data demonstrating that Bregs have a range of phenotypes, and there is not one single known Breg transcription factor. So—similarly to effector B cell subsets—Bregs may not be one distinct lineage [44], but could rather arise from multiple ‘precursors’ in response to local environmental factors [53].

### Polyfunctionality and heterogeneity of cytokine-producing B cell subsets: unresolved questions

The data discussed above demonstrate that human B cells can produce different cytokine profiles under various inflammatory conditions. Based on their impact on immune responses, B cells can be broadly separated into two functional groups. However, it is important to highlight there is significant plasticity within these functional groups, with reports over the last 40 years demonstrating that human B cells can often co-express IL-10 with the pro-inflammatory cytokines TNF and IL-6 [22, 62–64], and with type 2 cytokines such as IL-4 and IL-12 [27]. These data demonstrate that many cytokine-producing B cell subsets are polyfunctional. Furthermore, to date, no unique lineage marker or transcription factor has been identified that can distinguish between effector and regulatory B cells, or between effector B cell subsets themselves. Thus, it is difficult to define whether human B cells that produce different cytokine profiles are defined cellular states/fates, or functional states that arise in response to the different environmental stimuli. Recent data in the field suggest that the latter is more likely, and that this phenotypic heterogeneity is important in controlling the strength of cytokine-producing B cell responses. Indeed, the recent study executed by Glass and collaborators—which used mass cytometry to perform high dimensional characterisation of human B cells following activation in IL-10 skewing conditions—demonstrated that IL-10^+^ B cells can emerge from numerous B cell subsets. Dependent upon activation, distinct phenotypic profiles were associated with co-expression of IL-6/TNFα [62]. Notably, it is unclear whether a B cell that has previously produced for example IL-10, can ‘switch’ phenotypes to take on a pro-inflammatory role in another environment. Lastly, B cell that produce cytokines can also produce antibodies—as is the case for regulatory plasmablasts [41]—further highlighting the complex nature of this system.

The ability of some cytokine-producing B cell subsets to produce antibodies and to differentiate from both antigen-experienced (e.g. memory B cells and plasma cells) and non-antigen-experienced (e.g. transitional) B cells highlights another unresolved topic in this field: the role of antigen specificity in the induction of cytokine-producing B cells. Many studies in humans have identified BCR-crosslinking through stimulation with anti-IgM as a critical signal for the induction of certain cytokine profiles following B cell activation [6]. This suggests that the antigen specificity of B cells likely plays an important role in controlling cytokine production in at least some situations. Furthermore, data from the murine system have demonstrated that stimulation of isolated B cells with disease-inducing antigens, such as collagen type 2 in collagen-induced arthritis [37] or MOG in EAE [35], perpetuates IL-10 production by B cells, suggesting that antigen-specificity either directly or indirectly plays a role in Breg functionality. However, in humans, the exact role of BCR specificity in cytokine-producing B cell responses remains elusive. Some resolution on this important topic will likely be provided in the coming years, with the growing application of single cell RNA sequencing to different human tissue and disease cohorts, which allows co-analysis of BCR repertoire with cytokine gene expression. Thus, characterising the exact biological cues that define whether a B cell becomes pro- or anti-inflammatory, as well as investigating the antigen-specificity of these cells, remains a critical consideration for future research of human cytokine-producing B cell responses. These data likely have broad clinical applicability due to the growing appreciation of the central role that B cells play in the pathology of multiple disorders, and in particular autoimmune diseases where B cell depletion is used routinely in clinic with varying efficacy.

### Cytokine-producing B cell subsets in autoimmune disease and response to B cell depletion therapy

B cells were initially identified as key players in autoimmunity due to their ability to differentiate into autoantibody-producing plasma cells. However, it is now clear that B cells contribute to autoimmune inflammation via additional mechanisms, i.e. by presenting autoantigens to autoreactive T cells, through secreting pro-inflammatory cytokines, and through Breg dysfunction [2]. Accordingly, an imbalance between pro-inflammatory cytokine-producing B cells (mainly B cells producing IL-6) and IL-10-producing B cells has been described in multiple autoimmune diseases.

After the initial observation that the expansion in IL-6-producing B cells and reduction in IL-10-producing B cells are hallmarks of dysregulated cytokine networks in MS [23], Breg subsets are now well known as functionally and/or numerically deficient in multiple autoimmune diseases [43]. For example, Blair and colleagues noted that Bregs were functionally—but not numerically—deficient in patients with active SLE, a classic-driven autoimmune disease [8]. They depleted PBMCs from healthy donors and SLE patients of CD19^+^CD24^hi^CD38^hi^ B cells, before stimulating them with anti-CD3. In healthy donor samples, the absence of this Breg population led to a significant increase in the frequency of CD4^+^TNFα^+^ and CD4^+^IFNγ^+^ T cells compared with nondepleted PBMCs [8]. However, this effect was absent in cultures using PBMCs from patients with SLE [8]. They further found that CD19^+^CD24^hi^CD38^hi^ Bregs from SLE patients had a defective CD40 response, as STAT3 phosphorylation after CD40-activation was lower than in healthy Bregs, and IL-10 production was impaired [8]. In a later study, Menon and colleagues showed that the number of CD24^+^CD38^hi^ B cells negatively correlated with SLE disease activity, and that CD24^+^CD38^hi^ B cells from patients with SLE had a reduced capacity to suppress IFNα production by pDCs [59]. Interestingly, in this study—where IFNα was shown to increase IL-10 production by healthy B cells via STAT3 phosphorylation—stimulation of SLE B cells with IFNα did not lead to IL-10 upregulation, but rather to an increase in the production of IL-6 and TNFα by B cells. This altered cytokine response was associated with a defect in STAT3 phosphorylation and an increase in tSTAT1 [59].

The same group has also reported that RA patients with active disease have significantly lower frequencies and absolute numbers of CD19^+^CD24^hi^CD38^hi^ Bregs compared to healthy controls [61]. In this study, the number of CD19^+^CD24^hi^CD38^hi^ Bregs also negatively correlated with clinical and serological (as measured by C-reactive protein) disease activity in patients with RA [61]. Iwata and colleagues further explored IL-10^+^ B cell frequencies in 91 patients with several autoimmune diseases (SLE, RA, primary Sjögren’s syndrome, autoimmune vesiculobullous skin disease, or MS), and found that numbers were not significantly lower in these patients compared to healthy controls [10]. Upon stimulation with CpG and CD40L for 48 hours, the frequency of IL10^+^ B cells was significantly increased in all diseases compared to healthy controls [10]. Of note, most of the patients included in this study had zero or mild disease activity, which was controlled with or without medication (though disease activity information was not included for all patients) [10]. More recently, Piper and colleagues showed that—while stimulation with the TLR7 agonist R848 can induce both IL-10 and IL-6 by healthy B cells—B cells from patients with the childhood autoimmune disease juvenile dermatomyositis (JDM), have a defect in IL-10 production and an increase in IL-6 production [65]. Notably, this observed phenotype was context dependent, as the same imbalance in IL-6/IL-10 production was not observed when JDM B cells were stimulated with anti-CD40 [65].

The contribution of cytokine-producing B cells is critical when considering the now wide application of B cell depletion therapy (BCDT)—for example with anti-CD20 monoclonal antibodies (i.e. rituximab)—in autoimmune diseases including SLE, RA, and MS (albeit with varying efficacy). Even when controlling disease, BCDT has a limited impact on antibody-producing plasma cell frequency (as these cells do not express CD20) [66], illustrating that other pathogenic B cell mechanisms, such as cytokine production, are at play. It was recently hypothesised that depletion of IL-6-producing B effector cells plays a key role in the ability of BCDT to alleviate autoimmune conditions such as MS [67]. Accordingly, the abnormal levels of IL-6 produced by B cells from patients with MS are normalized by BCDT. Notably, the depletion of all CD20-expressing B cells also depletes the IL-10-producing regulatory B cell compartment. It has been suggested that the deficiency in IL-10 production associated with autoimmune conditions is ‘reset’ in patients with long-term response to BCDT, and that the repopulated B cells are biased towards favourable IL-10 production [59, 67]. Why BCDT leads to a ‘resetting’ of abnormal cytokine production in some patients and not in others remains a mystery. Interestingly, in a study using samples from patients with MS, it appeared that a microRNA-132-situin-1 axis contributed to dysregulated pro-inflammatory cytokine production by B cells. Treatment *in vitro* with pharmacological activation of situin-1, resveratrol, selectively normalised exaggerated production of TNFα and LT by MS B cells, while leaving the ability of these cells to produce IL-10 ‘intact’ [68]. This suggests that future therapeutic strategies could be developed that selectively target pro-inflammatory functions of B cells. This is an exciting prospect considering the adverse effects associated with Breg depletion that have been recently reported [69].

It is important to note that dysregulated cytokine networks within the B cell compartment have not only been described in autoimmunity. Key examples include the observation that patients with acute COVID-19 display an IL-6/IL-10 cytokine imbalance in response to Toll-like receptor activation [70], that allograft-tolerated liver transplant recipients have a higher proportion of IL-10 producing B cells compared to transplant-recipient controls [62], and that an expansion of IL-10-producing B cells in the periphery and within the tumour itself is associated with adverse outcomes in multiple cancers including breast, colorectal [47, 58] and gastric cancer [71]. It will be interesting for the field to observe if more selective B cell-targeted therapies are developed, whether they have clinical applicability beyond autoimmunity.

### Concluding remarks

In this review, we have summarised key studies demonstrating that human B cells produce a wide array of cytokines, and that B cell-derived cytokines play a significant role in immune responses. Following early observations in the 1980s and 1990s, the field has grown exponentially, demonstrating that depending on the B cell subtype and local environment—determined by other cells, such as T helper cells or dendritic cells, as well as cytokines and other proteins—B cells can produce both pro- and anti-inflammatory cytokines. Activation signals from CD4^+^ T cells can cause effector B cells to release pro-inflammatory cytokines—such as IL-6, TNFα, and IFNγ—which in turn further stimulate T cells. B cells can also regulate immunity, which they mainly do via the release of the anti-inflammatory cytokine IL-10. Notably, polyfunctional subsets of B cells also exist where B cells co-express both pro-inflammatory and anti-inflammatory cytokines. Different populations of cytokine-producing B cells have gained significant interest over the last decade due to the expanding evidence that differences in dysregulated B cell cytokine production can be correlated with different disease outcomes in multiple pathologies. In this review, we have focused on studies reporting dysregulated B cell cytokine production in autoimmune diseases due to the wide application of BCDT to these disorders in clinical practice. We have also summarised the growing evidence that the efficacy of B cell depletion in some patients is, at least in part, due to its ability to ‘repair’ defective B cell functions, such as impaired IL-10 production, even after repopulation. These data importantly highlight that the response to B cell-targeting therapies in autoimmune conditions remains heterogeneous, with some patients gaining long-term disease control and others flaring upon B cell repopulation. The development of more selective therapeutics, based on a greater understanding of what signals induce discrete effector functions in B cells, may therefore have greater efficacy for a larger number of autoimmune patients, and potentially in other disorders beyond autoimmunity.

Taken together, the studies summarised in this review demonstrate that B cells are not just antibody factories and that—considering the strong evidence of their potent immunomodulatory functions—B cell-derived cytokines can no longer be overlooked as a crucial part of both pro-and anti-inflammatory responses. A greater understanding of the molecular mechanisms controlling cytokine production by B cells and the diverse roles these cells play in human immune responses and immune-mediated pathology remains of critical importance. Further study of the induction, function, and stability of cytokine-producing B cell subsets is necessary to allow the full therapeutic potential of these cells to be harnessed for the benefit of human health.

## Data Availability

No data availability is required.

## Animal research guidelines

No animal research is presented in this paper.
